# An NDT Method for Measuring the Diameter and Embedment Depth of the Main Rebar in Cement Poles Based on Rotating Permanent Magnet Excitation

**DOI:** 10.3390/s25051477

**Published:** 2025-02-27

**Authors:** Hejia Wang, Lan Xiong, Zhanlong Zhang, Zhenyou Liu, Hanyu Yang, Hao Wu

**Affiliations:** 1 School of Electrical Engineering, Chongqing University, Chongqing 400044, China; 20183426@cqu.edu.cn (H.W.); lxiong@cqu.edu.cn (L.X.); lzy123@cqu.edu.cn (Z.L.); yanghanyu_1@163.com (H.Y.); 2 Chengdu High-tech Power Supply Branch, State Grid Sichuan Power Company, Chengdu 610000, China; wuha19910724@126.com

**Keywords:** cement pole, magnetic rotation, rebar diameter, rebar embedment depth, CNN-LSTM

## Abstract

Cement poles serve as supporting components for transmission lines and are widely used in medium- and low-voltage transmission networks. The main rebar is the primary load-bearing structure of the pole, and the accurate measurement of its diameter and embedment depth is crucial for quality control and safety assessment. However, existing non-destructive testing methods lack the accuracy of quantifying the internal main rebar of cement poles, and the measurement process is complex, cumbersome, and inefficient. To address this issue, this paper proposes a magnetic rotation-based detection method for measuring the diameter and embedment depth of the main rebar within cement poles. A specially designed H-type magnetic excitation structure is proposed, coupled with a detection technique utilizing rotating permanent magnets. The magnetic induction intensity data were acquired at seven distinct rotation angles using sensors, and the collected data were subsequently combined with a CNN-LSTM model to invert the diameter and embedment depth of the main rebar. The experimental results indicate that the method significantly improved the measurement accuracy compared with the condition of fixed magnetic excitation, with reductions in root mean square error (RMSE) of 46.71% and 35.57% for the diameter and embedment depth measurements, respectively. This method provides a robust, efficient, and accurate solution for quantifying the main rebar within cement poles, addressing the challenge associated with the quality assessment and health monitoring of these structures.

## 1. Introduction

Cement poles serve as crucial load-bearing components in distribution lines, enabling the long-distance transmission of electrical energy by the supporting conductors. Due to their extended service life and low maintenance costs, cement poles are widely used in medium- and low-voltage lines, with the 10 kV voltage class being the most commonly utilized type in power systems. However, the operation of cement poles in outdoor atmospheric conditions makes them prone to cracking of the concrete protective layer and corrosion of the rebar. Such cracking and damage can accelerate the corrosion process [1,2,3]. Corrosion not only reduces the effective cross-sectional area of the rebar but also weakens the bond stress between the concrete matrix and the rebar, diminishing the load-bearing capacity of the cement poles and potentially resulting in pole failures [4,5,6]. Therefore, the accurate measurement of the internal main rebar parameters in cement poles is crucial for health monitoring during their operational phase, evaluating the resilience to disasters (e.g., earthquakes [7]), and conducting post-disaster safety assessments. However, the precise detection of the embedment depth and diameter of the main rebar within the cement poles has continuously posed a significant challenge for maintenance and operation personnel.

In the case of cement poles, the term “main rebar” refers to the steel rebars arranged longitudinally along the interior of the pole, bearing the main load (such as bending moment, tensile stress). Currently, the methods for detecting the rebar structural parameters (e.g., diameter, embedment depth) are categorized into destructive testing (DT) and non-destructive testing (NDT). NDT enables structural assessment without compromising the integrity and has been extensively applied in the defect detection of metal wires [8,9], pipeline inspection [10], and internal rebar evaluation in concrete structures [11]. Given that DT methods inevitably damage the concrete components, leading to a premature loss of functionality of components and structures, the current research has predominantly focused on NDT techniques for internal rebar assessment [12,13,14]. Among the NDT approaches for rebar parameter detection, the most widely adopted methods include radiographic imaging, ultrasonic testing, ground-penetrating radar, and magnetic non-destructive testing. Dong et al. [15] utilized a high-resolution X-ray camera to acquire three-dimensional tomographic images of reinforced concrete samples, quantifying the cross-sectional area of the rebar and corrosion products along the height of the samples. However, the considerable size of the detection equipment posed challenges for on-site application, and the radiation emitted could negatively impact human health and the environment. In the study employing ultrasound for detecting the structural parameters of the internal rebar in concrete, Ruoyu et al. [16] developed a two-dimensional SH-FWI method that extracted information about the rebar’s position, concrete cover, and diameter from the analyses of the ultrasonic wave field; however, the measurement process required approximately 20 min. Suhaib et al. [17] employed planar wave imaging techniques to reconstruct the sub-surface images of reinforced concrete, extracting unit pixel contours that mapped the rebar features in the images to estimate the radius and central coordinates of objects such as pipes. Nevertheless, ultrasound technology frequently encounters issues related to ultrasonic scattering, leading to uncertainties in the measurement results [18]. Ground Penetrating Radar (GPR) is also a widely used non-destructive testing technique characterized by its high sensitivity to the embedment depth. Feng et al. [19] located rebars by extracting the peak coordinates from GPR reflection waveforms and roughly estimated the embedment thickness, subsequently employing a lookup database method to estimate the rebar diameter and embedment thickness based on electromagnetic induction intensity data. To address the challenge of accurately measuring the rebar diameter from radar waveforms, Cheng et al. [20] utilized a linear array of UWB antennas to obtain a full matrix of MIMO data regarding the rebar, reconstructing the rebar images using a diffraction stacking algorithm and measuring the rebar diameter based on the embedment depth and the 3 dB drop technique. However, the radar method is more suitable for detecting large-scale reinforced concrete frame structures and exhibits limitations when applied to the curved surface structures of cement poles.

The magnetic testing method has emerged as a popular non-destructive testing technique for rebars in recent years [21,22]. Research has indicated that variations in the structural parameters of rebars or the occurrence of corrosion lead to changes in the spatial distribution of the magnetic field, which in turn affects the output voltage of Hall sensors [23,24]. Gobov et al. [25] determined the embedment depth of rebars by measuring stray magnetic fields and their gradients, and derived the diameter using the normal stray magnetic field component and embedment depth. Frankowski et al. [26] designed a magnetic excitation system composed of two permanent magnets, fixing the magnets and scanning the sample with an AMR sensor along different directions on the surface of the concrete. This method effectively separated the amplitude, correlation, and offset of the magnetic field waveform, making measurements simpler and more accurate. However, the aforementioned two methods did not take into account the effects of adjacent parallel rebars, and still exhibited considerable errors when detecting rebars of certain specifications. Zhou et al. [27] proposed a magnetic-based detection method that required prior knowledge of both the main rebar diameter and the spacing between adjacent rebars to achieve high-precision inversion of the main rebar embedment depth, with an error of less than 0.5 mm. However, in practical production, the distribution of rebars in cement poles (i.e., rebar spacing) often deviates from the design specifications, and the main rebar diameter is typically not accurately known beforehand. If the assumed main rebar diameter, based on experience, significantly differs from the true value, the resulting embedment depth measurement errors will exceed the acceptable limits, making it difficult to achieve the claimed level of accuracy. Li et al. [28] utilized a trained GA-BP neural network model to measure the diameter and embedment depth of the internal main rebar in concrete poles, using the measured magnetic induction intensity data as the input. However, this method resulted in measurement errors of ±2 mm for the main rebar and ±3 mm for the embedment depth. Given that the diameter of the main rebar in 10 kV prestressed concrete poles is typically less than 12 mm, the relative error in the main rebar measurement could reach 16.67%, which is clearly insufficient to meet the high-precision measurement requirements. Furthermore, both of the aforementioned studies employed excitation coils as the magnetic excitation source. Excitation coils require a continuous external power supply to maintain the magnetic field, leading to a substantial increase in energy consumption, particularly during prolonged or large-scale inspections, and consequently increasing operational costs. In addition, Joule heating generated by prolonged energizing can cause temperature increases, potentially leading to aging or even damage to the coil insulation material, which can also affect the accuracy of the magnetic induction intensity measurements.

Compared to other detection methods, magnetic non-destructive testing (NDT) offers advantages such as a simple principle, low equipment costs, and safety for both humans and the environment. However, the current magnetic testing technique faces challenges in achieving accurate measurements of both diameter and embedment depth simultaneously, in addition to the complexity of the detection process. To address these issues and limitations, this study proposed a method for quantifying the main rebar in cement poles using rotating permanent magnets for excitation. This method utilized permanent magnets rather than excitation coils as the magnetic excitation. An H-type magnetic excitation structure was designed to enhance the sensitivity of magnetic induction intensity signal detection. Additionally, a novel detection approach that involved the rotation of the permanent magnets was introduced, along with an array of 12 Hall elements to collect spatial magnetic field data at various rotational angles. Furthermore, an intelligent inversion algorithm was employed to process the measurement data, enabling the precise inversion of the main rebar diameter and embedment depth. It provides a reliable, efficient, and precise tool for quality evaluation and health monitoring of cement poles, directly contributing to the development of safer and more sustainable transmission networks.

## 2. Detection Principle and Simulation Model Construction

### 2.1. Detection Principle

The body of the cement pole primarily consists of a rebar, concrete, and rusted rebar material. The presence of elemental iron in the rebar results in a relative permeability that is significantly greater than that of air. Similar to air, the relative magnetic permeability of both concrete and rusted rebar material is approximately 1 [29]. This characteristic renders the detection of the internal main rebar within the cement pole using external magnetic excitation a feasible approach.

**Figure 1:**
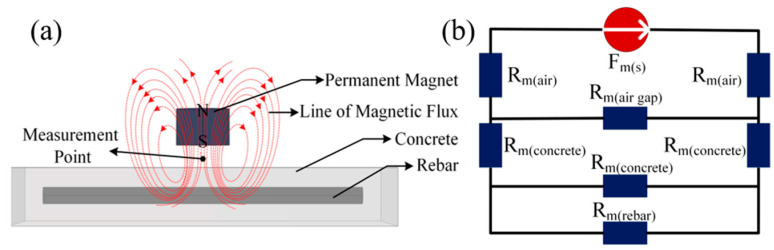
The schematic diagram of the magnetic detection method: (**a**) the path of the magnetic flux lines; (**b**) equivalent model of magnetic circuit.

**Figure 2:**
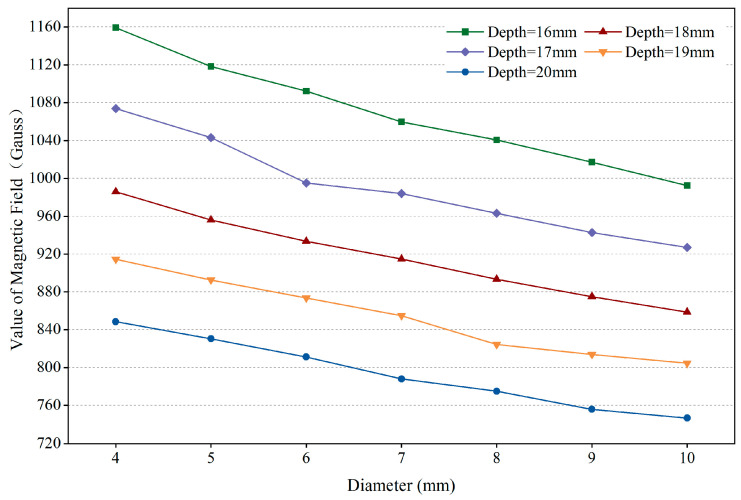
Magnetic induction intensity (B) at the measuring point versus the diameter and depth of the rebar.

In reinforced concrete structures, the main path for magnetic flux comprises a magnetic field source, ferromagnetic metals, air gaps, and concrete (as illustrated in Figure 1a). By altering the structural parameters of the ferromagnetic metals shown in the figure, the variations in magnetic field values at the specified measurement points (as depicted in Figure 1a) can be observed, as illustrated in Figure 2. Adopting the circuit analysis approach, we constructed an equivalent model of a magnetic circuit to analyze the reasons for the changes in magnetic induction intensity, where the magnetic potential $F_{m\left(s\right)}\;$ of the magnetic field source and the magnetic resistance $R_{m}$ in the magnetic circuit can be represented by Equation (1) to Equation (2):

(1) $$F_{m\left(s\right)}=H_{m}L_{m}$$

(2) $$R_{m}=\frac{L}{\mu_{0}\mu_{r}S}$$

where $H_{m}$ represents the self-demagnetizing field strength of the permanent magnet; $L_{m}$ indicates the length of the permanent magnet; *L* is the length of the magnetic conductor parallel to the magnetic field; $\mu_{0}$ is the vacuum permeability; $\mu_{r}$ is the relative permeability of the material; *S* signifies the cross-sectional area of the magnetic conductor perpendicular to the magnetic field. The equivalent model of the magnetic circuit is illustrated in Figure 1b. According to Equation (2), when the concentration of iron elements varies, $R_{m\left(rebar\right)}$ will change, subsequently affecting the magnetic flux lines through the rebar and air. Therefore, by calculating the magnetic induction intensity in the air, one can measure the structural parameters of the internal main rebar of the cement pole.

### 2.2. H-Type Magnetic Excitation Structure

**Figure 3:**
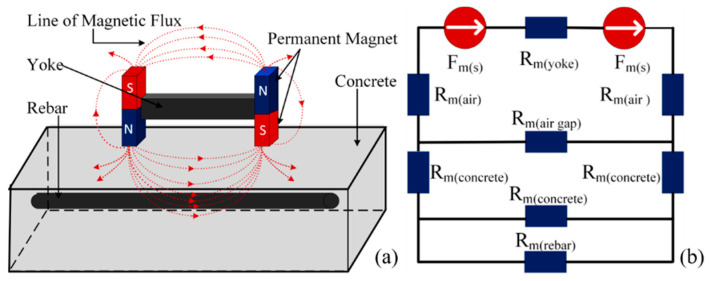
H-type structure of magnetic excitation: (**a**) schematic diagram of magnetic excitation; (**b**) equivalent model of magnetic circuit.

The magnetic reluctance of the rebar is significantly lower than that of the air, causing the magnetic flux lines to prefer flowing through the loop formed by the permanent magnet, iron yoke, air gap, and rebar. When a single permanent magnet is used as the magnetic excitation source, only a small segment of the magnetic flux lines passes through the rebar, while the majority propagate through the air, resulting in a limited magnetization effect of the permanent magnet on the rebar. To achieve better magnetization of the rebar, capture more pronounced magnetic induction intensity variation signals, and collect additional spatial magnetic induction intensity information, an H-type magnetic excitation structure was designed (as shown in Figure 3a). This structure consisted of two neodymium–iron–boron permanent magnets with the residual magnetic flux density of 1.2 T and an iron yoke made of DT4 pure iron, with its equivalent model of the magnetic circuit depicted in Figure 3b.

### 2.3. Equivalent Modeling of Cement Poles and Comparison of Different Magnetic Excitations

This study analyzed the performance of the H-type structure of magnetic excitation using finite element simulations. The detailed simulation models of the cement pole for the magnetic induction intensity calculation were built in COMSOL Multiphysics 6.2. The most common voltage level for distribution lines is 10 kV, and the primary types of cement poles at that voltage level are Z-∅190 × 12 × K × Y and Z-∅190 × 15 × M × G-III. The specific structural parameters and relative magnetic permeability of the main components are shown in Table 1 and Table 2.

**Figure 4:**
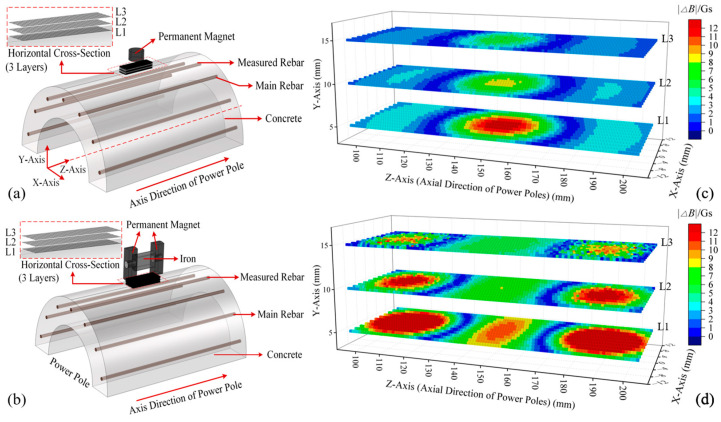
Equivalent model of cement pole and simulation results under different magnetic excitations: (**a**) a single permanent magnet and its corresponding pole model; (**b**) the H-type magnetic excitation and its corresponding pole model; (**c**) variations in the magnetic flux density across the spatial cross-section when parameters of the measured main rebar changed under the excitation of a single permanent magnet; (**d**) variations in the magnetic flux density across the spatial cross-section when parameters of the measured main rebar changed under H-type magnetic excitation.

Due to the slender design of the cement pole, its overall length reaches up to 15 m. In the calculations, the limitations in computer processing power prevented a detailed grid subdivision of the entire pole. Therefore, this study selected a section of the pole for refined modeling. Considering the “wick effect”, corrosion is likely to occur at approximately 1 m above the ground [33,34]. Consequently, a magnetic induction intensity calculation simulation model for the cement pole at this height was developed. In cement poles, the main rebars are the primary load-bearing structure, withstanding tensile and bending stresses. Compared to the main rebar, stirrups have a smaller radius and larger spacing, serving primarily to assist the main rebar in bearing loads and to maintain the pole’s diameter. Consequently, this paper mainly focuses on the structural parameters of the main rebar located between the stirrups, and therefore, the stirrups were excluded from the modeling process. Additionally, this research emphasized using the values of magnetic induction intensity to invert the diameter and embedment depth of the main rebars to assess their compliance. The influence of adjacent main rebars on the measured rebar is minor and diminishes with increasing spacing. When the distance reaches three main rebars from the measured rebar, its external magnetic field influence can be disregarded. Therefore, the number of main rebars can be reduced to simplify the simulation model, thereby enhancing the computational speed. The simulation model of the Z-∅190 × 12 × K × Y pole is shown in Figure 4a.

To facilitate the comparison, models were constructed for a single permanent magnet (Figure 4a) and an H-type magnetic excitation source (Figure 4b). For each model, three horizontal cross-sections (L1 to L3) were selected between the magnetic excitation source and the outer surface of the cement pole, aiming to investigate the changes in spatial magnetic induction intensity caused by the variations in the structural parameters (i.e., diameter and burial depth) of the measured main rebar. L1 is closest to the pole, located 5 mm from the outer surface of the cement pole, while L3 is nearest to the magnetic excitation source. The spacing between each cross-section is 5 mm. In both models, the embedment depth of the measured main rebar was increased by 2 mm. The changes in magnetic induction intensity at the three cross-sections were calculated, and the absolute values of these changes were obtained, with the results presented in Figure 4c,d. Under single permanent magnet excitation, the most significant change in magnetic induction intensity occurred at the center of section L1, directly beneath the magnetization surface of the cylindrical permanent magnet. The average change in magnetic induction intensity ($\Delta B_{ave}$) for each cross-section was calculated, yielding values of 4.25 Gs, 3.08 Gs, and 2.35 Gs for sections L1 to L3, respectively. When employing an H-type magnetic excitation, the most pronounced changes in magnetic induction intensity were observed at section L1, specifically at two locations: directly beneath the magnetization surfaces of the two permanent magnets and at the midpoint between them. The $\Delta B_{ave}$ values for sections L1 to L3 are 9.09 Gs, 6.27 Gs, and 4.69 Gs, respectively, which is approximately double the values observed under the previous condition. The analysis indicated that using the H-type magnetic excitation provided higher sensitivity when detecting the spatial magnetic flux density variations due to the changes in the structural parameters of the main rebar, and it captured effective magnetic induction intensity variation information over a larger spatial range.

**Figure 5:**
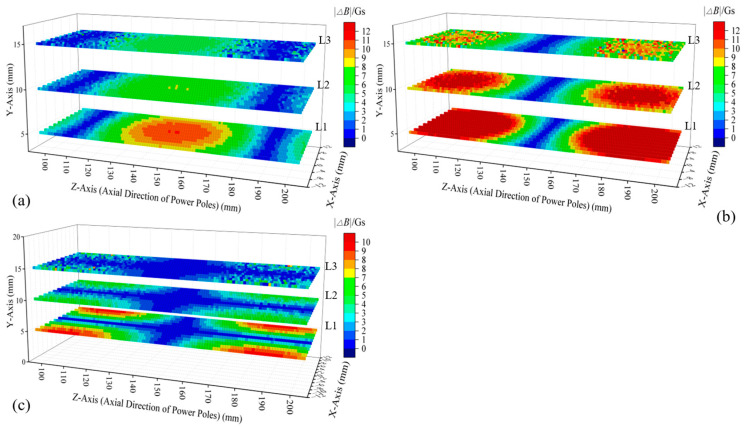
Absolute values of variations in magnetic induction intensity in different directions across three cross-sections under changed rebar embedment depth conditions: (**a**) *Z*-axis direction; (**b**) *Y*-axis direction; (**c**) *X*-axis direction.

Furthermore, in practical applications, twelve one-dimensional Hall sensors were arranged to measure the magnetic field values at twelve different points. According to the principle of the Hall effect, the change in the output signal of the Hall sensor is proportional to the variation in the magnetic flux density perpendicular to its plane. Since the distribution of magnetic flux lines is in three dimensions and the variations in magnetic field are more pronounced in the directions with higher flux density, the orientation and serial number of the designed Hall sensors are illustrated in Figure 5.

In this study, Hall sensors were implemented for measuring the magnetic induction intensity during the actual measurements. Given that the three-dimensional vector nature of magnetic induction intensity and the single-axis detection capability of Hall sensors (limited to the perpendicular field components relative to their working surfaces), the sensor array configuration was strategically optimized to maximize the sensitivity to the parameter-induced magnetic field variations through considering both the spatial position and magnetic field component direction. As depicted in Figure 4, the Cartesian coordinate system spanned X∈[−12, 12] mm and Z∈[96, 204] mm, with the magnetic excitation structure projecting onto X∈[−10, 10] mm and Z∈[100, 200] mm. The excitation assembly comprised two 20 mm permanent magnets and a 60 mm ferromagnetic yoke, partitioned into magnet regions (Z = 100~120 mm and 180~200 mm) and yoke region (Z = 120~180 mm), where the magnet regions were facing the magnetized surface of permanent magnets, and the yoke region was facing the yoke.

**Figure 6:**
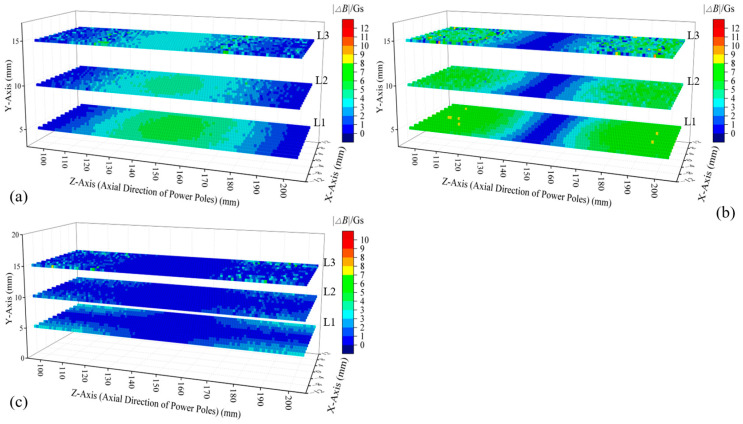
Absolute values of variations in magnetic induction intensity in different directions across three cross-sections under changed rebar diameter conditions: (**a**) *Z*-axis direction; (**b**) *Y*-axis direction; (**c**) *X*-axis direction.

Under constant main rebar diameter conditions, a 2 mm increase in embedment depth induced characteristic variations in the magnetic induction intensity components across three cross-sections, as illustrated in Figure 5. Conversely, with a fixed embedment depth, a 2 mm diameter increment generated variations in magnetic induction intensity components, shown in Figure 6. While absolute values of magnetic induction intensity variations differed between parameter changes, consistent patterns emerged:

① Yoke region (Z = 120–180 mm): *Z*-axis magnetic induction intensity variation amplitude exhibited proximity-enhanced sensitivity, peaking at the L1 cross-section with the maximum amplitude and broadest sensitive zone. See Figure 5a and Figure 6b.

② Magnet regions (Z = 100–120 mm, 180–200 mm): In general, the closer the cross-section was to the cement pole, the more significant the changes in the *Y*-axis component of magnetic induction intensity, both in terms of amplitude and the spatial extent of the sensitive area. See Figure 5b and Figure 6b.

③ The responses of the magnetic induction intensity in the *X*-axis direction to diameter changes were weaker, with significantly lower amplitude changes compared to other directions. See Figure 5c and Figure 6c.

The L1 cross-section was selected for optimal sensor deployment. Considering the physical volume limitations of the sensors, which require at least a 10 mm spacing between adjacent sensors, the final array arrangement was as follows:

① Yoke region (Z = 120–180 mm): At Z = 145 mm and Z = 155 mm, three measurement points were arranged along the *X*-axis direction with a 10 mm spacing, used to calculate the magnetic field component in the *Z*-axis direction.

② Magnet regions (Z = 100–120 mm, 180–200 mm): At the positions directly facing the center of the magnetization surface of the permanent magnets (Z = 110 mm, 190 mm), three measurement points were arranged along the *X*-axis direction with a 10 mm spacing, used to calculate the magnetic field component in the *Y*-axis direction.

**Figure 7:**
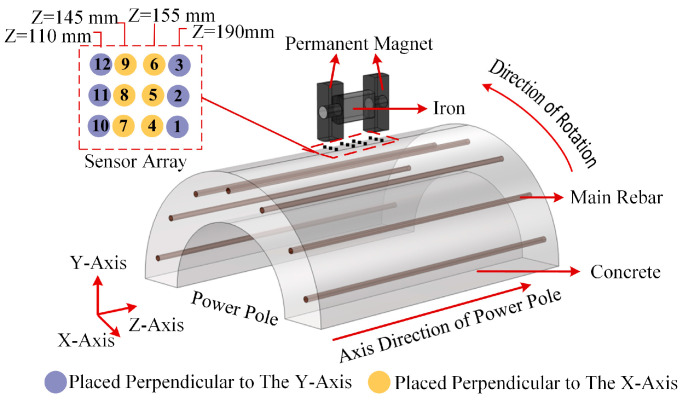
Serial number of sensors and its mounting direction.

A total of 12 coordinate positions were selected as measurement points and used to calculate the magnetic field components in the *Z*-axis and *Y*-axis directions, as shown in Figure 7.

## 3. Test Methods for the Main Rebar Parameters

When the magnetic excitation was positioned far from the internal main rebar of the cement pole, the sensors encountered difficulties in detecting the spatial magnetic induction intensity variations induced by the changes in the structural parameters of the main rebar. To effectively collect the spatial magnetic induction intensity information necessary for inverting the diameter and embedment depth of the main rebar, the distance between the magnetic excitation device and the main rebar needed to be minimized, which entailed positioning it directly opposite the main rebar. Therefore, the accurate localization of the main rebar was essential before proceeding with the measurements of its diameter and embedment depth.

### 3.1. Peak-Valley-Based Method for Locating the Main Rebar

In the previously established magnetic induction intensity calculation model for the cement pole, the magnetic excitation device and the points characterizing the installation positions of the Hall sensors were rotated along the circumferential direction of the external surface of the cement pole (as shown in Figure 7), and the magnetic induction intensity values at the corresponding points were calculated. The results are presented in Figure 6a,b.

**Figure 8:**
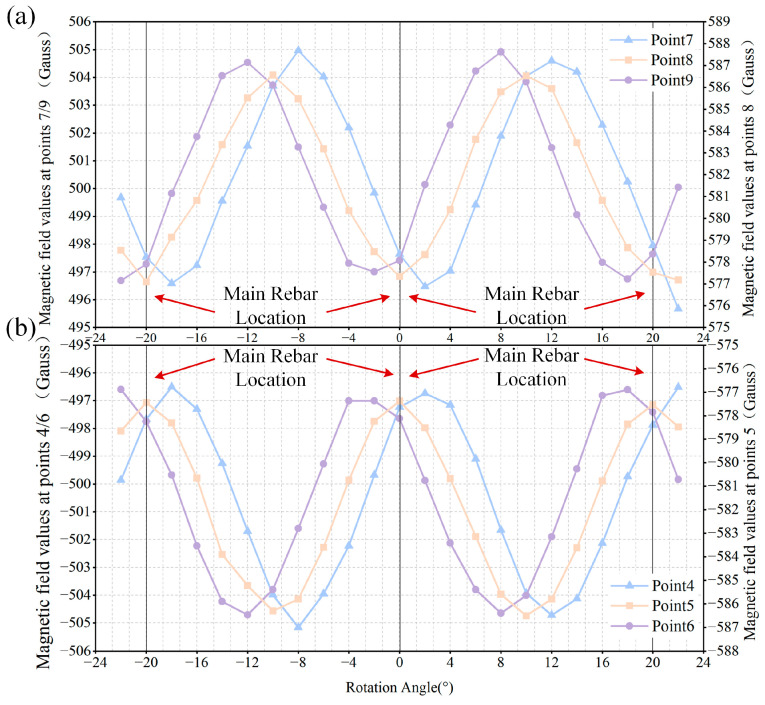
Data of magnetic flux density obtained from sensors: (**a**) magnetic flux density acquired from sensor 7 to sensor 9; (**b**) magnetic flux density acquired from sensor 4 to sensor 6.

According to the curves presented in Figure 8, the magnetic induction intensity value at point 5 reached its peak when rotated to the midpoint between the two main rebars, while the value at point 8 corresponded to a trough. Conversely, when positioned directly opposite the center of the main rebars, the results were reversed. Thus, by marking the positions of the peaks and troughs, one can effectively determine both the center and orientation of the main rebars.

### 3.2. Rotation Design for Magnetic Excitation

During the process of collecting magnetic induction intensity data using an H-type magnetic excitation device, two primary issues were identified: First was the inherent zero drift of Hall sensors. When the sensor output unexpectedly deviates from the true zero point over time, the output signal continually exhibits bias, thereby affecting the accuracy of the measurement results. Second, the inversion of the internal main rebar structural parameters (specifically, diameter and embedment depth) from spatial magnetic induction intensity values inherently exhibits ill-posed characteristics within the mathematical framework of inverse problems. Additional methods, such as introducing additional magnetic excitation, adjusting the conditions of magnetic excitation application, and increasing the number of sensors, are necessary to capture more magnetic field information. Furthermore, considerations regarding the size and weight limitations of the detection equipment, as well as the principle of minimizing human operational errors, must also be addressed.

**Figure 9:**
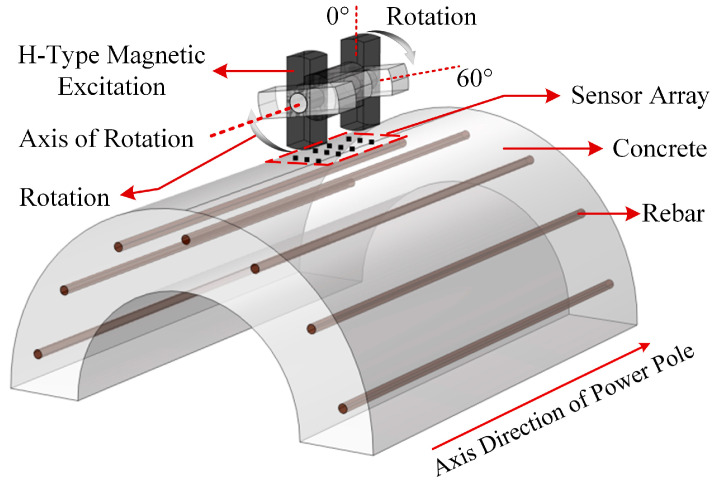
Schematic diagram of magnetic excitation rotation.

To resolve these two issues, a method involving rotational magnetic excitation was proposed for the first time. After the positioning of the main rebar was completed, the positions of sensors were fixed while the magnetic excitation was driven by a motor to rotate around the axis of rotation, as illustrated in Figure 9. To address the sensor zero-drift problem, an additional compensation mechanism was introduced to determine the absolute zero value of the sensor (i.e., the current drift value of the sensor). Firstly, two permanent magnets were aligned with the magnetization surface facing the cement pole, at which point the sensor measurements were represented by matrix $a_{1}$. Secondly, the magnetic excitation structure rotated 180° around the central axis of the magnetic yoke to switch the magnetic pole, and the measured magnetic induction intensity values of sensors were represented by matrix $a_{2}$. Finally, the matrix *c*, which contained the drift information of each sensor, could be calculated based on $a_{1}$ and $a_{2}$, and the element values in *c* were set to the corresponding absolute zero values of the sensors, as detailed in Equations (3)–(5).

(3) $$a_{1}=b+c$$

(4) $$a_{2}=-b+c$$

(5) $$c=\frac{\left(a_{1}+a_{2}\right)}{2}=\frac{\left(b+c-b+c\right)}{2}$$

where matrix *b* represented the true values of the spatial magnetic induction intensity. By taking the negative of the values of each element within matrix *b*, denoted as *−b*, the true values of the spatial magnetic induction intensity after the magnetic pole switching could be obtained. The elements within matrix *c* represented the offset values of the magnetic induction intensity measurements taken by the sensors (absolute zero values). Subtracting the corresponding absolute zero values from the sensor measurements allowed for the acquisition of the true values of the magnetic induction intensity.

During the rotation of the magnetic excitation, the relative position between the magnetic excitation and the cement pole changed, thereby altering the conditions under which the magnetic excitation is applied. By collecting the spatial magnetic induction intensity values based on these changes, the quantity of features inputted into the algorithm can be enriched, thereby improving the inversion accuracy of the main rebar’s diameter and embedment depth. However, as the angle between the normal of the magnetization surface of the permanent magnets and the radial normal of the cement pole gradually increased, the magnetic flux lines passing through the main rebar decreased, diminishing the magnetization effect on the rebar. This led to subtle changes in the spatial magnetic induction intensity resulting from the variations in the structural parameters of the main rebar. Furthermore, collecting excessive magnetic induction intensity at different rotation angles may result in a lengthy feature sequence being fed into the algorithm, not only causing feature redundancy but also giving rise to the “curse of dimensionality”, which complicated and hindered the training of the algorithm model. Therefore, it was necessary to select appropriate rotation angles for the magnetic excitation to ensure that significant magnetic induction intensity changes were induced by the variations in the structural parameters of the main rebar under specific conditions.

We selected the appropriate rotation angles for the magnetic excitation through simulation and computational analysis. Firstly, based on the relevant parameters of cement poles Z-∅190 × 12 × K × Y and Z-∅190 × 15 × M × G-III, we established 14 simulation models for the magnetic induction intensity of the cement poles, designated as Models 1 to 8 and Models 9 to 14. The parameters for the diameter and embedment depth of the main rebar within each model are presented in Table 3.

In each model, the magnetic excitation was configured to rotate from −16° to 16° in 2° increments, resulting in a total of 17 rotation angles. The values of magnetic induction intensity were calculated for 12 sensors at these angles. To visually illustrate the variations in the magnetic flux density with different structural parameters of the main rebars, the computed magnetic flux density was processed. Let $A_{\left(p\right)ij}$ and $A_{\left(q\right)ij}$ represent the magnetic flux densities obtained from the *i*-th sensor when the magnetic excitation rotated to the *j*-th angle in the *p*-th and *q*-th models, respectively. In Model 1 to Model 8, the magnetic flux density calculated from Model 1 serves as the reference value; in Model 9 to Model 14, the magnetic flux density calculated from Model 9 serves as the reference value. Therefore, we can obtain:

(6) $$\left\{\begin{array}{l} e_{\left(p\right)ij}=\left|A_{\left(p\right)ij}-A_{\left(1\right)ij}\right|,p=2,\ldots ,8\\ e_{\left(q\right)ij}=\left|A_{\left(q\right)ij}-A_{\left(9\right)ij}\right|,q=10,\ldots ,14 \end{array}\right.$$

where $e_{\left(p\right)ij}$ represents the difference between two magnetic flux densities, minuend is the magnetic flux density at the *i*-th sensor in the *p*-th model when the magnetic excitation rotated to the *j*-th angle, and subtrahend is the corresponding magnetic flux density from Model 1. Similarly, $e_{\left(q\right)ij}$ denotes the difference between the magnetic flux density at the *i*-th sensor in the *q*-th model rotated to the *j*-th angle and the corresponding magnetic flux density from Model 9. Consequently, a 12-row by 17-column matrix can be generated. To visually illustrate the intensity of variation in the magnetic flux density at each sensor’s location, $e_{\left(p\right)ij}$ and $e_{\left(q\right)ij}$ underwent normalization:

(7) $$\left\{\begin{array}{l} e_{\left(p\right)j,\max}=\max\left(e_{\left(p\right)1j},e_{\left(p\right)2j},\ldots ,e_{\left(p\right)12j}\right),p=2,\ldots ,8,j=1,2,\ldots ,17\\ e_{\left(q\right)j,\max}=\max\left(e_{\left(q\right)1j},e_{\left(q\right)2j},\ldots ,e_{\left(q\right)12j}\right),q=10,\ldots ,14,j=1,2,\ldots ,17 \end{array}\right.$$

(8) $$\left\{\begin{array}{l} e_{\left(p\right)ij}^{\prime }=\frac{e_{\left(p\right)ij}}{e_{\left(p\right)j,\max}}\\ e_{\left(q\right)ij}^{\prime }=\frac{e_{\left(q\right)ij}}{e_{\left(q\right)j,\max}} \end{array}\right.$$

**Figure 10:**
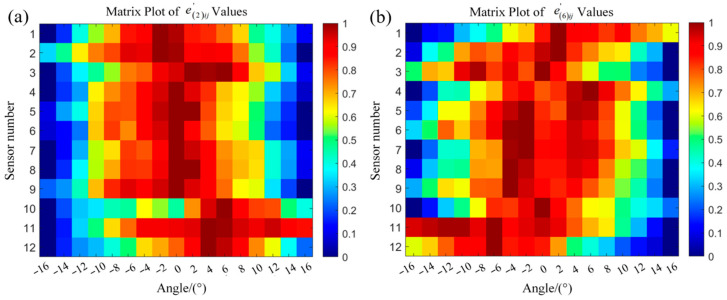
Color matrix diagrams of the normalized values reflecting the variation in the magnetic induction intensity across the different models: (**a**) matrix plot of $e_{\left(2\right)ij}^{\prime }$; (**b**) matrix plot of $e_{\left(6\right)ij}^{\prime }$.

Taking Model 2 and Model 6 as examples, these two models represent cases with variations in the embedment depth and diameter of the main rebars. After normalizing $e_{\left(2\right)ij}$ and $e_{\left(6\right)ij}$ according to Equations (7) and (8), the resulting matrices $e_{\left(2\right)ij}^{\prime }$ and $e_{\left(6\right)ij}^{\prime }$ reflect the intensity of variation in the magnetic flux density at 12 sensors across various angles, as illustrated in Figure 10.

**Figure 11:**
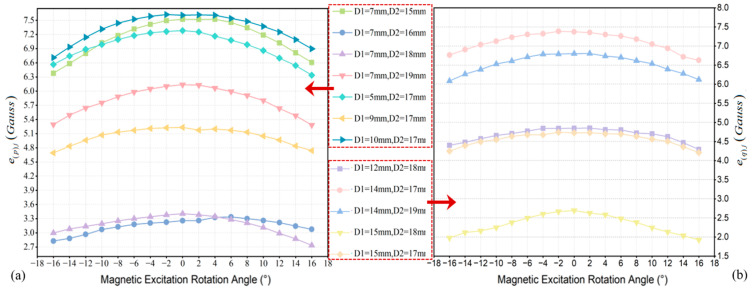
Mean absolute deviation of the sensors when the parameters of the main rebar were changed at different angles of the magnetic excitation: (**a**) calculated $e_{\left(p\right)j}$ for Model 2 to Model 8; (**b**) calculated $e_{\left(q\right)j}$ for Model 10 to Model 14.

Figure 10a,b illustrates that the horizontal axis represents the angle of rotation of the magnetic excitation, while the vertical axis denotes the sensor identification numbers. Overall, when the parameters of the measured main rebars changed, the variations in the magnetic flux density measured at the sensor locations became more pronounced as the magnetic excitation device rotated between −6° and 6°. Further processing of the magnetic field variation data $e_{\left(p\right)ij}$ and $e_{\left(q\right)ij}$ was conducted.

(9) $$\left\{\begin{array}{l} e_{\left(p\right)j}=\sum_{i=1}^{12}\frac{\left|e_{\left(p\right)ij}\right|}{12},p=2,\ldots ,8,j=1,2,\ldots ,17\\ e_{\left(q\right)j}=\sum_{i=1}^{12}\frac{\left|e_{\left(q\right)ij}\right|}{12},q=10,\ldots ,14,j=1,2,\ldots ,17 \end{array}\right.$$

where both $e_{\left(p\right)j}$ and $e_{\left(q\right)j}$ represent the mean absolute deviation, which quantified the intensity of magnetic flux density variations as the parameters of the main rebars changed with different angles of the magnetic excitation. $e_{\left(p\right)j}$ is defined as the average value calculated by summing the absolute values of the $e_{\left(p\right)ij}$ corresponding to the 12 sensors in the p-th model, which were computed using the magnetic excitation at the *j*-th angle. Similarly, $e_{\left(q\right)j}$ was calculated in the same manner. After processing the data from various models, Figure 11a,b were obtained, where D1 represents the diameter and D2 indicates the embedment depth.

Figure 9 clearly illustrates that the overall magnetic flux density variations were more pronounced when the parameters of the main rebars changed, as the angle of rotation of the magnetic excitation transitions from −6° to 6°. Therefore, during the detection process, the angles of rotation for the magnetic excitation were set sequentially to −6°, −4°, −2°, 0°, 2°, 4°, and 6°.

## 4. Inversion and Detection of the Diameter and Embedment Depth of the Main Rebar

### 4.1. Detection Experiments and Dataset Collection

**Figure 12:**
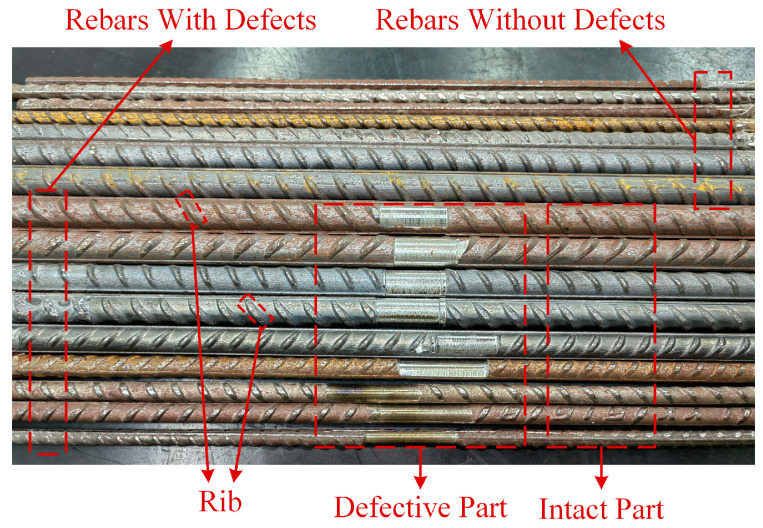
Part graph of the rebars used for the experiment.

**Figure 13:**
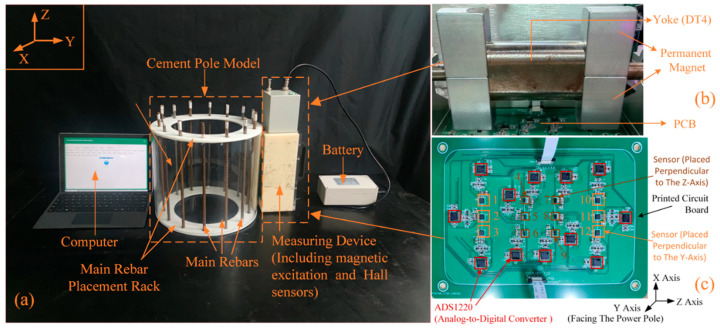
Schematic diagram of the experiment platform: (**a**) acrylic rack with 14 rebars and data acquisition system; (**b**) picture of H-type magnetic excitation; (**c**) picture of PCB with sensor array.

The surface of the rebars actually used inside concrete poles is not smooth but features fine ribs (see Figure 12). Therefore, we collected data using experimental methods conducted in the laboratory. To simulate different diameters and embedment depths of the main rebars within the concrete poles, we constructed dozens of main rebar placement racks with varying geometric parameters through 3D printing technology. The material of the racks is acrylic, which has the same relative magnetic permeability as that of concrete [35]. A schematic diagram of the measurement setup is illustrated in Figure 13.

In the data collection phase, to comprehensively consider the different working conditions of the main rebars on site, this study constructed two types of datasets:

(a) The first type consisted of measured data collected under conditions where the main rebars exhibited no significant defects. In the study area, 10 kV power transmission lines primarily utilize prestressed cement poles, which feature relatively small main rebar diameters. Consequently, when constructing the dataset, the range for the main rebar diameters was set from 5.1 mm to 13.6 mm, and the embedment depth range was established from 13.5 mm to 22.5 mm, totaling 300 sets (where the embedment depth refers to the distance from the outer surface of the concrete pole to the surface of the rebars).
(b) The second type comprised measured data collected under conditions where the main rebars displayed significant corrosion defects. To simulate the corrosion of the main rebars, a milling machine was used to shave off parts of the rebars. In practice, the highest concentration of chloride ions on the surface of the rebars was found closest to the concrete cover, while the lowest concentration was located furthest from the concrete cover, resulting in non-uniform corrosion of the rebars [23,36,37]. Hence, shaving was performed only on one side of the main rebars to simulate the remaining effective portion after corrosion (see Figure 10). The minimum diameter of the processed main rebars (i.e., the intact portion diameter minus the maximum depth of the defect) ranged from 4.1 mm to 12.8 mm, while the embedment depth remained between 13.5 mm and 22.5 mm, resulting in a total of 250 sets of this type of data.

Ultimately, data samples were collected, with each data sequence consisting of 84 measurements from 12 sensors at 7 different angles of magnetic excitation (−6°, −4°, −2°, 0°, 2°, 4°, and 6°). These 550 data samples were subsequently divided into training and testing sets at an 80:20 ratio for model training and evaluation.

### 4.2. Construction of the Inversion Model

Long Short-Term Memory (LSTM) networks are an enhanced version of Recurrent Neural Networks (RNNs) that effectively mitigate the vanishing and exploding gradient problems commonly associated with traditional RNNs, making them particularly adept at handling sequential data [38]. In this study, the LSTM was designed as a single-layer structure, consisting of a hidden layer with 100 units and utilizing the hyperbolic tangent (tanh) activation function. However, a single LSTM network is prone to overfitting; thus, integrated LSTM networks composed of LSTM layers/networks combined with other components, such as Convolutional Neural Networks (CNNs) and external memory units, have emerged [39]. Convolutional Neural Networks (CNNs) are multilayer neural networks comprising input layers, convolutional (CONV) layers, activation function layers, pooling layers, and fully connected (FC) layers, with the convolutional and pooling layers being the most critical components [40]. The convolutional layers are employed to extract data features, whereas the pooling layers reduce the number of parameters in the model and help prevent overfitting.

**Figure 14:**
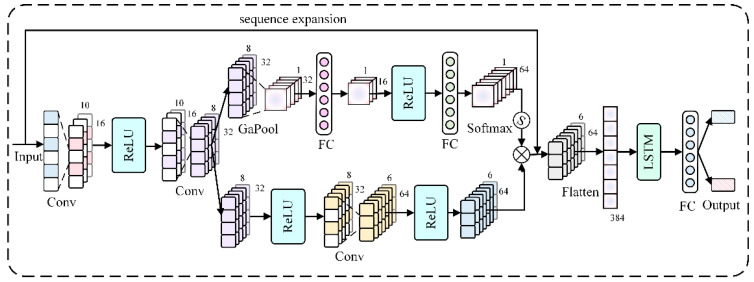
Structure of convolutional neural networks–long short-term memory.

In this paper, the CNN-LSTM network was utilized to invert the diameter and embedment depth of the main rebars. Initially, a sequence of length 84 was inputted, and convolution operations of CNNs were performed to extract the intricate features from the sequential data. Subsequently, the extracted higher-order features were processed through the LSTM layer. Finally, the estimated diameter and embedment depth values were produced through the fully connected layer. The CNN architecture in this study included three convolutional layers with 16, 32, and 64 convolutional kernels, respectively. The kernel size was set at 3 × 1, and a stride of 1 was employed alongside one pooling layer for global pooling operations. The specific structure of the CNN-LSTM model for inverting the diameter and embedment depth of main rebars is illustrated in Figure 14.

### 4.3. Results of the Inversion Experiments

The data collected in the previous subsection were divided into training and testing sets in a ratio of 4:1. Specifically, 440 sets of data were initially used to train the CNN-LSTM model, followed by the input of 110 sets of magnetic field data from the testing set into the model to obtain the inversion results for diameter and embedment depth, as illustrated in Figure 13a,b. For comparative analysis, magnetic field data collected at different rotation angles were also inputted into the model, including the following:

(a) Single angle: magnetic excitation without rotation;
(b) Three angles: magnetic excitation data collected at −2°, 0°, and 2°;
(c) Five angles: magnetic excitation data collected at −4°, −2°, 0°, 2°, and 4°.

**Figure 15:**
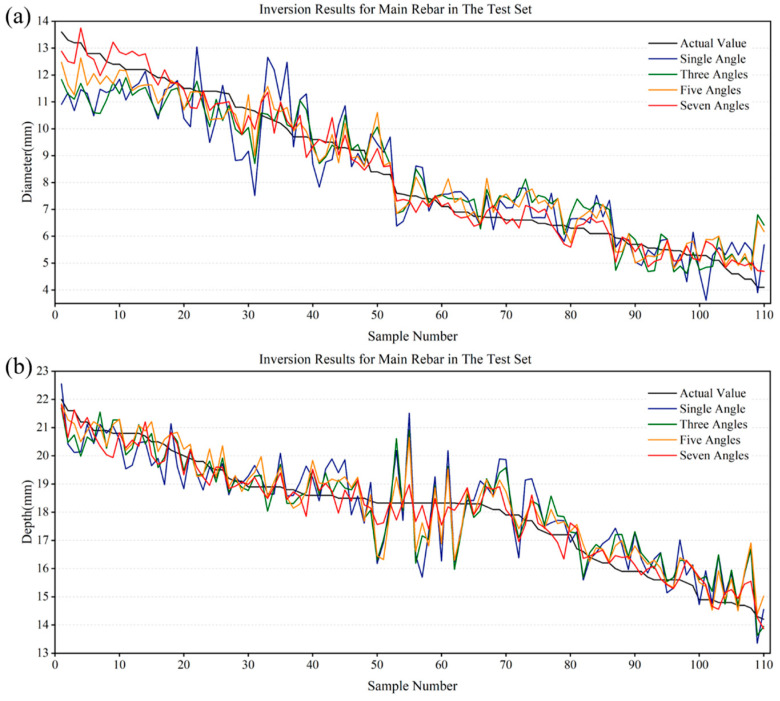
Inversion results: (**a**) diameter of the main rebars; (**b**) embedment depth of the main rebars.

Figure 15 shows that when the measured values of sensors were collected with magnetic excitation at seven rotation angles, and the values were used as inputs for the inversion algorithm, the output values for the 110 data in the testing set closely matched the true values. Specifically, the maximum inversion error for the diameter was 1.1 mm, and the maximum inversion error for the embedment depth was 0.95 mm, with 73 data items having inversion errors for both diameter and embedment depth not exceeding 0.50 mm. In terms of inversion results, when the magnetic excitation was rotated by seven angles, the overall precision for both the diameter and embedment depth was superior to scenarios involving no rotation of magnetic excitation (single angle), three angles, and five angles. Notably, compared to the data collection with magnetic excitation without rotation, the accuracy of the inversion for the diameter and embedment depth of the main rebars within the cement poles was significantly improved.

To comprehensively evaluate the inversion performance for the diameter and embedment depth, four commonly used metrics were selected: Root Mean Square Error (*RMSE*), Mean Absolute Error (*MAE*), R-Squared (*R^2^*), and Mean Absolute Percentage Error (*MAPE*). The specific calculation formulas are as follows:

(10) $$RMSE=\sqrt{\frac{\sum_{i=1}^{n}{\left(y\left(i\right)-\overset{\wedge }{y}\left(i\right)\right)}^{2}}{n}}$$

(11) $$MAE=\frac{\sum_{i=1}^{n}\left|y\left(i\right)-\overset{\wedge }{y}\left(i\right)\right|}{n}$$

(12) $$R^{2}=1-\frac{\sum_{i=1}^{n}{\left(y\left(i\right)-\overset{\wedge }{y}\left(i\right)\right)}^{2}}{\sum_{i=1}^{n}{\left(y\left(i\right)-\bar{y}\left(i\right)\right)}^{2}}$$

(13) $$MAPE=\frac{100\%}{n}\sum_{i=1}^{n}\left|\frac{y\left(i\right)-\overset{\wedge }{y}\left(i\right)}{y\left(i\right)}\right|$$

**Figure 16:**
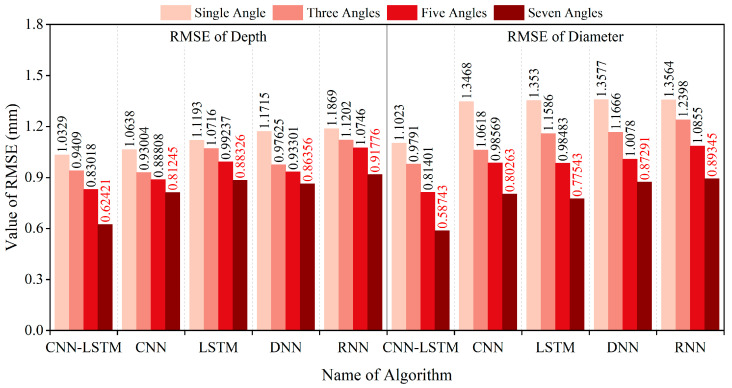
RMSE values of diameter and embedment depth inversion results for different models.

**Figure 17:**
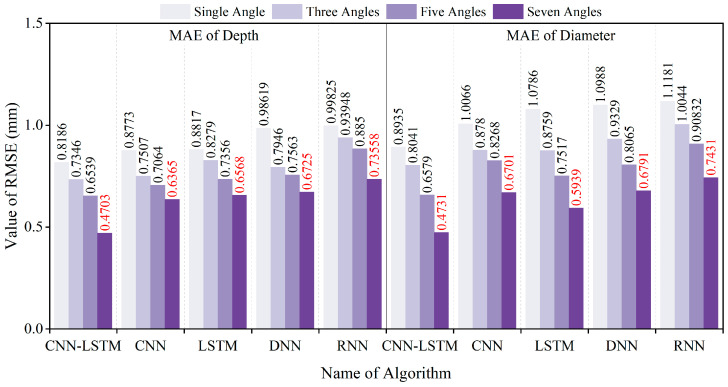
MAE values of diameter and embedment depth inversion results for different models.

**Figure 18:**
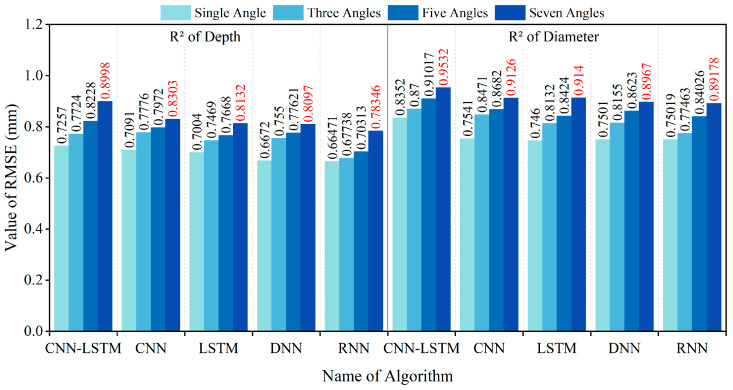
R^2^ values of diameter and embedment depth inversion results for different models.

**Figure 19:**
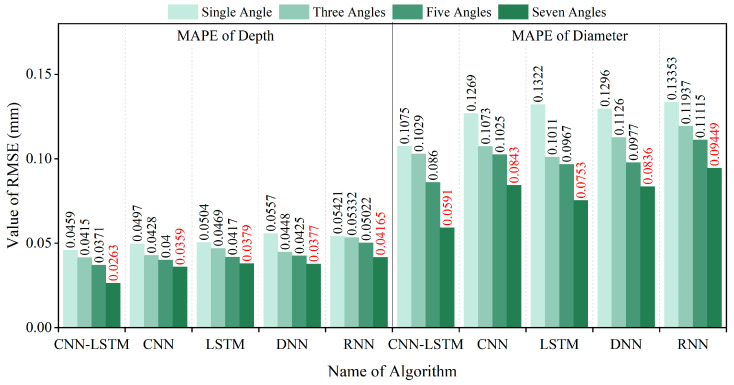
MAPE values of diameter and embedment depth inversion results for different models.

In this study, *n* represents the sample size, while $y\left(i\right)$*,* $\hat{y}\left(i\right)$*, and* $\bar{y}\left(i\right)$ denote the actual value, inversion value, and average value of the i-th sample, respectively. The smaller the *RMSE*, *MAE*, and *MAPE* values, and the larger the *R^2^* value, the smaller the overall error, indicating a better inversion result. The analysis of inversion errors resulting from the use of different magnetic excitation rotation methods is illustrated in Figure 16, Figure 17, Figure 18 and Figure 19.

The dataset was also divided into training and testing sets using an 80:20 ratio. The CNN, LSTM, DNN, and RNN algorithms were trained using the training sets, and subsequently, the testing set was inputted into each of these models. The resulting *RMSE*, *MAE*, *MAPE*, and *R^2^* error analysis results are presented in Figure 16, Figure 17, Figure 18 and Figure 19.

The above results indicate that the accuracy of diameter and embedment depth inversion was significantly improved by increasing the number of magnetic excitation rotation angles. In terms of diameter inversion, when comparing the case of no rotation to that with seven rotation angles, the RMSE, MAE, and MAPE decreased by 46.71%, 47.05%, and 45.02%, respectively, while the R^2^ value increased by 14.13%. For embedment depth inversion, the RMSE, MAE, and MAPE values decreased by 39.57%, 42.55%, and 42.70%, respectively, with the R^2^ value increasing by 23.99% when rotating seven rotation angles. Furthermore, the CNN-LSTM model demonstrates superior performance over four alternative models through comprehensive comparative analysis. Quantitative validation reveals significant improvements in the inversion accuracy: Compared to standalone LSTM, CNN-LSTM achieved 24.25% and 29.33% reductions in RMSE for diameter and embedment depth inversion, respectively. Compared to the RNN algorithm, the CNN-LSTM reduced the RMSE for the diameter and embedment depth by 34.25% and 31.98%, respectively. This outcome substantiates LSTM’s enhanced capability over basic RNNs in processing long sequential data. In comparison to the CNN algorithm, the CNN-LSTM reduced the RMSE for the diameter and burial depth by 26.81% and 23.17%, respectively. When compared to the DNN algorithm, the CNN-LSTM reduced the RMSE for diameter and burial depth by 32.70% and 27.72%, respectively.

To further evaluate the robustness of the CNN-LSTM model for inverting the main rebar diameter and embedment depth, Gaussian white noise was strategically introduced as synthetic data perturbations. Gaussian white noise is a common type of random noise signal characterized by a Gaussian probability distribution, uniform power spectral density, and independent sample points that conform to a normal distribution. In our experiments, we found that environmental and temporal variations had a minimal impact on the sensor-detected background magnetic field values (i.e., the magnetic induction intensity values in the absence of ferromagnetic materials), with variations not exceeding 2 Gauss. Therefore, when adding Gaussian noise to the test data, we incorporated Gaussian noise with amplitudes within 2 Gauss, conforming to a normal distribution, to investigate its impact on the main rebar diameter and burial depth inversion results. After adding noise, the RMSE for diameter inversion was 0.76051, and the RMSE for burial depth was 0.79622. Although these results represent a decrease in the inversion accuracy compared to the case without added noise, the precision remains superior to the results obtained using the other four alternative models without noise, demonstrating a degree of robustness.

## 5. Conclusions

To address the challenge of accurately determining the structural parameters of the main rebar within cement poles, this study proposed a measurement method for the diameter and embedment depth of the main rebar based on rotating permanent magnet excitation. A measurement device was developed to collect magnetic induction intensity information with varying rotation angles of the magnetic excitation, and a deep learning model was introduced to achieve precise inversion of the main rebar’s diameter and embedment depth. The main conclusions are as follows:

(a) In response to the low detection sensitivity associated with using a single permanent magnet for excitation, a method using permanent magnets with an H-type structure as excitation was proposed. Simulation results indicated that the H-type magnetic excitation demonstrated superior magnetization effects and higher sensitivity in detecting spatial magnetic induction intensity variations compared to a single permanent magnet, thereby enabling the acquisition of effective magnetic induction intensity variation information across a larger spatial range.
(b) To overcome the issue of insufficient magnetic induction intensity information for accurately inverting the diameter and embedment depth of the main rebar in a stationary state, a method involving rotating magnetic excitation was introduced, with the appropriate rotation angles determined. This method not only calibrated the zero-point drift of the sensors but also collected additional spatial magnetic field values for the inversion of the main rebar’s diameter and embedment depth. The experimental results showed that collecting the magnetic induction intensity at seven rotation angles significantly improved the accuracy of the inversion of the main rebar parameters compared to fixed magnetic excitation. Furthermore, the use of the CNN-LSTM algorithm during inversion significantly outperformed the results obtained from the CNN, LSTM, and DNN algorithms.

This method has significant implications for quality control and the safety assessment of cement poles, effectively ensuring that the mechanical strength and durability of in-service poles meet the required standards, thereby guaranteeing the continuous and stable operation of the power transmission network. Since this study primarily focuses on the main rebar within 10 kV poles, the framework can be extended to assess the rebar parameters (diameter and embedment depth) in poles of varying voltage ratings and specifications. For regions employing main rebars with divergent magnetic properties, it is possible to quantify the region-specific main rebars of cement poles by performing transfer learning. Furthermore, the method provides a transferable analytical framework for detecting the geometric parameters of rebars in reinforced concrete structures, such as shear walls and bridge piers, which significantly improves the efficiency of the early diagnosis of anomalous structures, supports accurate preventive maintenance decision making, and ultimately realizes the synergistic optimization of the safety and service life of the entire lifecycle of building infrastructures. However, it is important to note that the proposed measurement method was conducted away from the area of the stirrups in the cement poles. Currently, the effective measurement of the parameters of the main rebars where they overlap with the stirrups remains a challenge. In the future, we may employ magnetic tomography techniques with an optimized multi-sensor array configuration to capture the magnetic field distribution in cross-sections. This will enable the reconstruction of a two-dimensional magnetic image of the concrete pole’s cross-section, facilitating the detection of the diameter and embedment depth of the main rebar in areas near the stirrups.

## Figures and Tables

**Table 1:** Specific structural parameters of 10 kV cement poles.

Type	Z-∅190 × 12 × K × Y	Z-∅190 × 15 × M × G-III
Inner diameter at the root end (mm)	230	290
Outer diameter at the root end (mm)	350	390
Inner diameter at the tip (mm)	80	90
Outer diameter at the tip (mm)	190	190
Pole height (mm)	12,000	15,000
Burial depth of the pole (mm)	2000	1500
Diameter of the rebar (mm)	17	18
Embedment depth of the rebar (mm)	7	14

**Table 2:** Relative permeability of the medium material [30,31,32].

Medium Material	Relative Magnetic Permeability
DT4	5000
Rebar	150
Air	1.00000037
Iron (III) oxide	1.0072
Concrete	1
Water	0.999992

**Table 3:** Diameter and embedment depth of the main rebar specified from different models.

Drawings Referenced	Model Number	Rebar Diameter (mm)	Embedment Depth (mm)
Z-∅190 × 12 × K × Y	Model 1	7	17
	Model 2	7	15
	Model 3	7	16
	Model 4	7	18
	Model 5	7	19
	Model 6	5	17
	Model 7	9	17
	Model 8	10	17
	Model 9	14	18
Z-∅190 × 15 × M × G-III	Model 10	12	18
	Model 11	14	17
	Model 12	14	19
	Model 13	15	18
	Model 14	15	17

## Data Availability

Data are contained within the article.

## References

[1] Kliukas R., Daniūnas A., Gribniak V., Lukoševičienė O., Vanagas E., Patapavicius A. (2017). Half a Century of Reinforced Concrete Electric Poles Maintenance: Inspection, Field-Testing, and Performance Assessment. Struct. Infrastruct. Eng..

[2] Cao C., Cheung M.M.S., Chan B.Y.B. (2013). Modelling of Interaction between Corrosion-Induced Concrete Cover Crack and Steel Corrosion Rate. Corros. Sci..

[3] Zhao Y., Dong J., Wu Y., Jin W. (2016). Corrosion-Induced Concrete Cracking Model Considering Corrosion Product-Filled Paste at the Concrete/Steel Interface. Constr. Build. Mater..

[4] Cai Y., Zhang W., Yu L., Chen M., Yang C., François R., Yang K. (2020). Characteristics of the Steel-Concrete Interface and Their Effect on the Corrosion of Steel Bars in Concrete. Constr. Build. Mater..

[5] Wu Y.-Z., Lv H.-L., Zhou S.-C., Fang Z.-N. (2016). Degradation Model of Bond Performance between Deteriorated Concrete and Corroded Deformed Steel Bars. Constr. Build. Mater..

[6] Zhao Y., Karimi A.R., Wong H.S., Hu B., Buenfeld N.R., Jin W. (2011). Comparison of Uniform and Non-Uniform Corrosion Induced Damage in Reinforced Concrete Based on a Gaussian Description of the Corrosion Layer. Corros. Sci..

[7] Vailati M., Monti G., Bianco V. (2021). Integrated Solution-Base Isolation and Repositioning-for the Seismic Rehabilitation of a Preserved Strategic Building. Buildings.

[8] Sun Y., Wu J., Feng B., Kang Y. (2014). An Opening Electric-MFL Detector for the NDT of In-Service Mine Hoist Wire. IEEE Sens. J..

[9] Xiao Y., Xiong L., Zhang Z., Dan Y. (2023). A Novel Defect Detection Method for Overhead Ground Wire. Sensors.

[10] Yuksel V., Tetik Y.E., Basturk M.O., Recepoglu O., Gokce K., Cimen M.A. (2023). A Novel Cascaded Deep Learning Model for the Detection and Quantification of Defects in Pipelines via Magnetic Flux Leakage Signals. IEEE Trans. Instrum. Meas..

[11] Hu J.Y., Zhang S.S., Chen E., Li W.G. (2022). A Review on Corrosion Detection and Protection of Existing Reinforced Concrete (RC) Structures. Constr. Build. Mater..

[12] Hassani S., Dackermann U. (2023). A Systematic Review of Advanced Sensor Technologies for Non-Destructive Testing and Structural Health Monitoring. Sensors.

[13] Zhang J., Peng L., Wen S., Huang S. (2024). A Review on Concrete Structural Properties and Damage Evolution Monitoring Techniques. Sensors.

[14] Kot P., Muradov M., Gkantou M., Kamaris G.S., Hashim K., Yeboah D. (2021). Recent Advancements in Non-Destructive Testing Techniques for Structural Health Monitoring. Appl. Sci..

[15] Dong B., Fang G., Liu Y., Dong P., Zhang J., Xing F., Hong S. (2017). Monitoring Reinforcement Corrosion and Corrosion-Induced Cracking by X-Ray Microcomputed Tomography Method. Cem. Concr. Res..

[16] Chen R., Tran K.T., Dinh K., Ferraro C.C. (2022). Evaluation of Ultrasonic SH-Waveform Tomography for Determining Cover Thickness and Rebar Size in Concrete Structures. J. Nondestruct. Eval..

[17] Reyaz S.U., Beniwal S., Ganguli A. (2024). Application of Plane Wave Imaging and Processing for Measurement of Reflectors in Concrete. IEEE Trans. Instrum. Meas..

[18] Abdelhafeez M., Nassr A.A., Abdelraheem M. (2021). Capacitance-Based Technique for Detection of Reinforcement Bars in Concrete Structures. IEEE Sens. J..

[19] Zhou F., Chen Z., Liu H., Cui J., Spencer B.F., Fang G. (2018). Simultaneous Estimation of Rebar Diameter and Cover Thickness by a GPR-EMI Dual Sensor. Sensors.

[20] Cheng W., Sun H.-H., Tan K.H., Fan Z. (2023). Estimating the Diameter of Reinforcing Bars Using an Ultra-Wideband MIMO GPR Array. Constr. Build. Mater..

[21] Eslamlou A.D., Ghaderiaram A., Schlangen E., Fotouhi M. (2023). A Review on Non-Destructive Evaluation of Construction Materials and Structures Using Magnetic Sensors. Constr. Build. Mater..

[22] Shilar F.A., Ganachari S.V., Patil V.B., Yunus Khan T.M., Saddique Shaik A., Azam Ali M. (2024). Exploring the Potential of Promising Sensor Technologies for Concrete Structural Health Monitoring. Materials.

[23] Fu C., Huang J., Dong Z., Yan W., Gu X.-L. (2020). Experimental and Numerical Study of an Electromagnetic Sensor for Non-Destructive Evaluation of Steel Corrosion in Concrete. Sens. Actuators A Phys..

[24] Li Z., Jin Z., Gao Y., Zhao T., Wang P., Li Z. (2020). Coupled Application of Innovative Electromagnetic Sensors and Digital Image Correlation Technique to Monitor Corrosion Process of Reinforced Bars in Concrete. Cem. Concr. Compos..

[25] Gobov Y.L., Mikhailov A.V., Smorodinskii Y.G. (2018). Magnetic Method for Nondestructive Testing of Rebar in Concrete. Russ. J. Nondestruct. Test..

[26] Frankowski P.K., Chady T. (2023). Evaluation of Reinforced Concrete Structures with Magnetic Method and ACO (Amplitude-Correlation-Offset) Decomposition. Materials.

[27] Zhou J., Qiu K., Deng B., Zhang Z., Ye G. (2022). A NDT Method for Location and Buried Depth Measurement of Rebars in Concrete Pole. IEEE Trans. Instrum. Meas..

[28] Li X., Ma R., Wu H., Dong Z., Wang Y., Dai Y. Research on Quality Inspection and Imaging Method of Rebar in Concrete. Proceedings of the 2022 4th International Symposium on Smart and Healthy Cities (ISHC).

[29] Mosharafi M., Mahbaz S.B., Dusseault M.B., Vanheeghe P. (2020). Magnetic Detection of Corroded Steel Rebar: Reality and Simulations. NDT E Int..

[30] Mahbaz S., Dusseault M.B., Cascante G., Vanheeghe P. (2017). Detecting Defects in Steel Reinforcement Using the Passive Magnetic Inspection Method. J. Environ. Eng. Geophys..

[31] Zhang J., Liu C., Sun M., Li Z. (2017). An Innovative Corrosion Evaluation Technique for Reinforced Concrete Structures Using Magnetic Sensors. Constr. Build. Mater..

[32] Ye H., Zhang Z., Dan Y., Gan P., Deng J., Pan Z. (2021). Novel Method for Measurement of Rebar State of Cement Tower. IEEE Trans. Instrum. Meas..

[33] Masoodi R., Pillai K.M. (2010). Darcy’s Law-Based Model for Wicking in Paper-like Swelling Porous Media. AIChE J..

[34] Baghmisheh A.G., Mahsuli M. (2021). Seismic Performance and Fragility Analysis of Power Distribution Concrete Poles. Soil. Dyn. Earthq. Eng..

[35] Zbojovský J., Kurimský J., Kolcunová I., Pavlík M., Cimbala R. Waveguide Model for the Purposes of Evaluating the Shielding Effectiveness of the Electromagnetic Field. Proceedings of the 2022 IEEE 5th International Conference and Workshop Óbuda on Electrical and Power Engineering (CANDO-EPE).

[36] Jin L., Liu M., Zhang R., Du X. (2020). Cracking of Cover Concrete Due to Non-Uniform Corrosion of Corner Rebar: A 3D Meso-Scale Study. Constr. Build. Mater..

[37] Wang F., Xue X., Hua J., Chen Z., Huang L., Wang N., Jin J. (2022). Non-Uniform Corrosion Influences on Mechanical Performances of Stainless-Clad Bimetallic Steel Bars. Mar. Struct..

[38] Afshari S.S., Zhao C., Zhuang X., Liang X. (2023). Deep Learning-Based Methods in Structural Reliability Analysis: A Review. Meas. Sci. Technol..

[39] Yu Y., Si X., Hu C., Zhang J. (2019). A Review of Recurrent Neural Networks: LSTM Cells and Network Architectures. Neural Comput..

[40] Dao F., Zeng Y., Qian J. (2024). Fault Diagnosis of Hydro-Turbine via the Incorporation of Bayesian Algorithm Optimized CNN-LSTM Neural Network. Energy.

